# Association of the *ACTN3* Gene’s Single-Nucleotide Variant Rs1815739 (R577X) with Sports Qualification and Competitive Distance in Caucasian Athletes of the Southern Urals

**DOI:** 10.3390/genes14081512

**Published:** 2023-07-25

**Authors:** Olga V. Balberova, Natalia A. Shnayder, Evgeny V. Bykov, Yuri E. Zakaryukin, Marina M. Petrova, Irina A. Soloveva, Ekaterina A. Narodova, Galina A. Chumakova, Mustafa Al-Zamil, Azat R. Asadullin, Elena E. Vaiman, Vera V. Trefilova, Regina F. Nasyrova

**Affiliations:** 1 Research Institute of Olympic Sports, Ural State University of Physical Culture, 454091 Chelyabinsk, Russia; bev58@yandex.ru (E.V.B.); yora6482@mail.ru (Y.E.Z.); 2 Institute of Personalized Psychiatry and Neurology, V.M. Bekhterev National Medical Research Center for Psychiatry and Neurology, 192019 St. Petersburg, Russia; vaimanelenadoc@gmail.com (E.E.V.); vera.v.trefilova@yandex.ru (V.V.T.); nreginaf77@gmail.com (R.F.N.); 3 Shared Core Facilities Molecular and Cell Technologies, V.F. Voino-Yasenetsky Krasnoyarsk State Medical University, 660022 Krasnoyarsk, Russia; stk99@yandex.ru (M.M.P.); solovieva.irina@inbox.ru (I.A.S.); katya_n2001@mail.ru (E.A.N.); 4 Department of Therapy and General Medical Practice with a Course of Additional Professional Education, Altai State Medical University, 656038 Barnaul, Russia; g.a.chumakova@mail.ru; 5 Department of Physiotherapy, Faculty of Continuing Medical Education, Peoples’ Friendship University of Russia, 117198 Moscow, Russia; alzamil@mail.ru; 6 Department of Psychiatry and Addiction, Bashkir State Medical University, 450008 Ufa, Russia; droar@yandex.ru; 7 International Centre for Education and Research in Neuropsychiatry, Samara State Medical University, 443016 Samara, Russia

**Keywords:** sports genetics, candidate genes, *ACTN3*, single nucleotide variant, skeletal muscles, athlete

## Abstract

An elite athlete’s status is associated with a multifactorial phenotype depending on many environmental and genetic factors. Of course, the peculiarities of the structure and function of skeletal muscles are among the most important characteristics in the context of athletic performance. Purpose: To study the associations of SNV rs1815739 (C577T or R577X) allelic variants and genotypes of the *ACTN3* gene with qualification and competitive distance in Caucasian athletes of the Southern Urals. Methods: A total of 126 people of European origin who lived in the Southern Urals region took part in this study. The first group included 76 cyclical sports athletes (speed skating, running disciplines in track-and-field): SD (short distances) subgroup—40 sprinters (mean 22.1 ± 2.4 y.o.); LD (long distances) subgroup—36 stayer athletes (mean 22.6 ± 2.7 y.o.). The control group consisted of 50 healthy nonathletes (mean 21.4 ± 2.7 y.o.). We used the Step One Real-Time PCR System (Applied Biosystems, USA) device for real-time polymerase chain reaction. Results: The frequency of the major allele R was significantly higher in the SD subgroup compared to the control subgroup (80% vs. 64%; *p*-value = 0.04). However, we did not find any significant differences in the frequency of the R allele between the athletes of the SD subgroup and the LD subgroup (80% vs. 59.7%, respectively; *p*-value > 0.05). The frequency of the X allele was lower in the SD subgroup compared to the LD subgroup (20% vs. 40.3%; *p*-value = 0.03). The frequency of homozygous genotype RR was higher in the SD subgroup compared to the control group (60.0% vs. 34%; *p*-value = 0.04). The R allele was associated with competitive distance in the SD group athletes compared to those of the control group (OR = 2.45 (95% CI: 1.02–5.87)). The X allele was associated with competitive distance in the LD subgroup compared to the SD subgroup (OR = 2.7 (95% CI: 1.09–6.68)). Conclusions: Multiplicative and additive inheritance models demonstrated that high athletic performance for sprinters was associated with the homozygous dominant genotype 577RR in cyclical sports athletes of Caucasian origin in the Southern Urals.

## 1. Introduction

It is generally accepted that an elite athlete’s status is associated with a multifactorial phenotype that depends on many environmental and genetic factors. Undoubtedly, the features of the structure and function of skeletal muscles are one of the most important characteristics in the context of sports performance in cyclic sports associated with the development of power/strength or endurance phenotypes [1].

Each person has an individual muscle composition. That is, the inherent combination of muscle cells (fibers) of different types in all skeletal muscles is unique to him. Moreover, the number of muscle fibers and the number of motor units (motoneuron, axon and the totality of innervated muscle fibers) in a person are genetically determined and remain unchanged throughout his entire life [2].

At the same time, deoxyribonucleic acid (DNA) nucleotide sequence variations, in particular SNVs, act as key internal factors (biomarkers) that are associated with high performance in sports [3]. Determining the genetic predisposition in specific individuals makes it possible to identify athletes who have the greatest genetically determined potential for certain sports that require the manifestation of speed, strength or endurance. Skeletal muscle composition and function are controlled by many different genes, and their SNVs can serve as biomarkers of the status of strength or endurance of athletes [4].

In this regard, in recent years, the number of studies focused on the attempts to establish an association between certain SNVs of candidate genes with a change in the functional activity of proteins and enzymes of muscle fibers that affect the individual response of the athlete’s body to the training process has increased exponentially in cyclic sports [5,6,7,8,9,10]. At least 155 genetic biomarkers (located in the nuclear DNA on almost all chromosomes and mitochondrial DNA) associated with an elite athlete’s status have been identified (93 genetic markers associated with endurance and 62 genetic markers associated with power/strength) [6]. These genetic biomarkers include SNV rs1815739 of the *ACTN3* gene (α-actinin skeletal muscle isoform 3). This SNV (C577T or R577X, rs1815739) of the *ACTN3* gene leads to the replacement of the amino acid arginine (R) with the stop-codon (X) and chain termination in the α-actin-3 protein [4,11,12].

**Figure 1:**
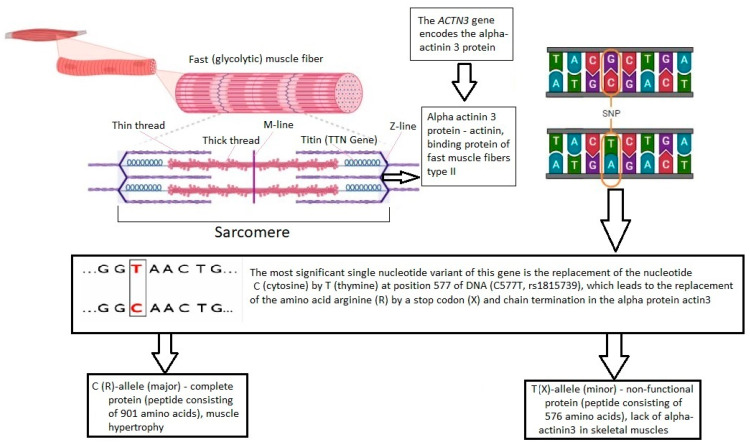
Scheme of a genetically determined dysfunction of actinin-binding protein in type II fast muscle fibers associated with a SNV rs1815739 of the *ACTN3* gene.

**Figure 2:**
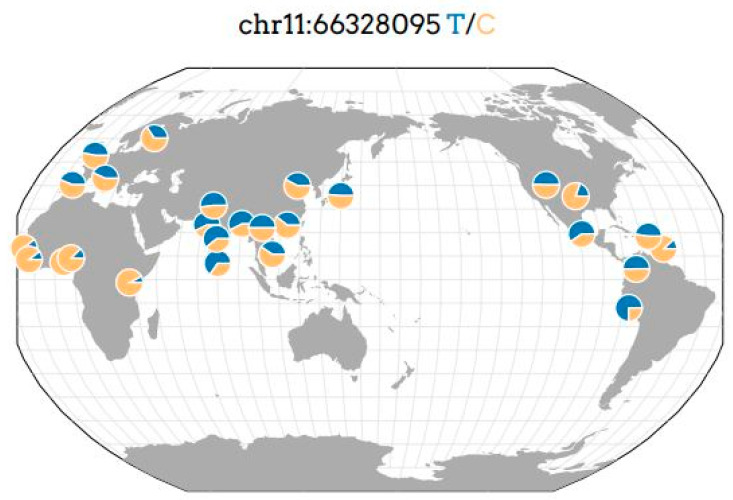
Geography of genetic variant rs1815739 of the *ACTN3* gene in the world [15].

The *ACTN3* gene determines the expression of the α-actinin-3 protein in skeletal muscle fibers, which cross-links and stabilizes thin actin filaments on the Z-disk (Figure 1). Therefore, the *ACTN3* gene is fundamental to produce strong and fast muscle contractions and explosive movements. [13]. The frequency of occurrence of a minor homozygous genotype can reach 16% in the world population [14]. However, the frequency of occurrence of the minor allele X (rs1815739) of the *ACTN3* gene is variable in different regions of the world (Figure 2), which can affect the variable sports performance of athletes in cyclic sports depending on their ethnic group and region of residence. The expression of α-actinin-3 is found only in type II fast fibers [15]. The α-actinin-3 deficiency in the carriers of the recessive homozygous XX genotype does not cause muscle disease due to the expression of highly homologous α-actinin-2 in all muscle fiber types [13,16]. Although α-actinin-2 and α-actinin-3 are almost identical and originate from repetitive gene duplication events, they are thought to play different roles in the skeletal muscle [12]. An abnormal (nonfunctional) α-actinin-3 protein in the carriers of the recessive homozygous XX genotype leads to the formation of an athlete phenotype, which is characterized by lower muscle strength and reduced muscle mass growth under the influence of the training process [7,8,17]. There is evidence of an association of this genotype with a decrease in the skeletal muscle mass, which persists into old age [12] with an increased risk of sarcopenia, weakness and loss of function in the elderly [13]. However, this genotype/phenotype is associated with an increase in the metabolic efficiency of skeletal muscle and the proportion of slow twitch type I muscle fibers. This is associated with an increase in the endurance of athletes in cyclic sports (for example, long-distance runners) [14].

Purpose—to study the associations of single-nucleotide variant rs1815739 (C577T) allelic variants and genotypes of the *ACTN3* gene with qualification and competitive distance in Caucasian athletes of the Southern Urals.

## 2. Materials and Methods

### 2.1. Data Collection

This study was part of the research work “Individualization of the training of a sports reserve based on the improvement of methods for controlling the functional state of athletes, taking into account genetic factors (on the example of the basic sports of the Chelyabinsk region)”. This study was supported by the state task of the Ministry of Sports of the Russian Federation (registration number AAAA-A16-116042510005-7 dated 25 April 2016) and was approved by the local ethics committee of the Ural State University of Physical Culture (UralSUPC), Protocol No. 5 dated 11 January 2016. This study protocol was in accordance with Declaration of Helsinki for Human Research—ethical principles of medical research with the participation of people, proclaimed at the 64th General Assembly of the World Medical Association (Fortaleza, Brazil, 2013) [18].

All athletes and healthy volunteers (nonathletes) signed a voluntary informed consent to participate in this study. Athletes and healthy volunteers (nonathletes) were not rewarded for participating in this study. All diagnostic procedures were performed free of charge for the participants in this study. This study was conducted on the basis of the Research Institute of Olympic Sports UralSUPC (Chelyabinsk, Russia) from January 2016 to December 2019.

Statistical processing and clinical interpretation of the results of this study was carried out based on the Research Institute of Olympic Sports (Chelyabinsk, Russia) in conjunction with the Institute of Personalized Psychiatry and Neurology of the V.M. Bekhterev National Medical Research Center for Psychiatry and Neurology (St. Petersburg, Russia) and the Shared Core Facilities “Molecular and Cellular Technologies” of the V.F. Voino-Yasenetsky Krasnoyarsk State Medical University (Krasnoyarsk, Russia) from January 2020 to June 2022.

Study type: open, observational, cross-sectional, randomized, case-control.

### 2.2. Participants

This study involved 126 participants of European origin living in the Southern Urals region (Chelyabinsk city and Chelyabinsk region, Russia). The first group included 76 athletes of cyclic sports (speed skating, track and field disciplines), including SD subgroup of 40 athletes—sprinters (sprint)—mean age 22.1 ± 2.4 years, and LD subgroup of 36 athletes—long-distance racers—mean age 22.6 ± 2.7 years. The control group consisted of 50 healthy nonathletes (volunteers), mean age 21.4 ± 2.7 years. The observation groups were comparable in terms of sample size, sex and age of the participants.

Criteria for inclusion to and criteria for exclusion from this study are presented in Table 1.

The sports title Master of Sports of International Class is awarded to an athlete who has set or confirmed a world or European record in sports disciplines included in the program of the Olympic Games. This record must be registered by international sports federations in the relevant sport, and the athlete must play for the sports team of the Russian Federation.

The sports title Master of Sports of Russia is awarded to an athlete who has won a prize in national or international competitions.

Sports category Candidate for Master of Sports and first adult category (1st Category) are assigned to athletes based on the results of performances at official sports competitions included in the Unified calendar plan of interregional, all-Russian and international sports events. The athlete must fulfill the qualification requirements for the assignment of the relevant qualification categories approved by the federal executive body in the field of physical culture and sports in the Russian Federation.

### 2.3. Genetic Analysis

Biological samples are buccal epithelium. They were taken using a cervical brush (cytobrush). All participants were informed that before collecting the buccal epithelium. It is necessary to refrain from eating for two hours. Immediately before taking the biomaterial, the subjects thoroughly rinsed their mouths with warm water. Samples were separated by centrifugation. The resulting aliquots were placed in a refrigerator and kept until DNA profiling at −18 °C.

Thus, a biobank of samples of DNA of athletes and nonathletes was created on the basis of the Research Institute of Olympic Sports (Chelyabinsk). Genomic DNA was extracted according to a standard protocol based on the clinical diagnostic laboratory of the Olympic Sports Research Institute (Chelyabinsk) [19].

Allelic variants and genotypes of the SNV rs1815739 (C577T) of the *ACTN3* gene on chromosome 11q13.2 were determined in all groups of participants by real-time polymerase chain reaction (RT-PCR) using diagnostic equipment Rotor-Gene 6000 (Corbett Life Science, Sydney, Australia) and technology allelic discrimination of TaqMan and fluorescent probes (Applied Biosystems, Waltham, MA, USA). In addition, 20% of the DNA samples were tested twice with 100% agreement of the results to ensure the control of the accuracy of PCR diagnostics.

The DNA testing consisted of the following components: 2.5× reaction mix for RT-PCR, which included a pair of allele-specific TaqMan MGB probes (comprising a DNA minor-groove-binding (MGB) fragment at the 3′ end and an integrated NFQ quencher); deoxynucleoside triphosphates; PCR buffer; MgCl2), Taq DNA polymerase with enzymeinhibiting antibodies and ultra-pure water. For DNA elution, buffers were added to the tubes, resuspended and incubated in a thermostat for 5 min at 65 °C with periodical vortexing. To remove the sorbent freed from DNA, the tubes were centrifuged for 1 min at a rotation speed of 12,000 rpm. The supernatant containing DNA was transferred into clean labeled tubes and stored at −20 °C.

Primers were designed according to the Applied Biosystems protocol (USA).

The C (R) allele, for rs1815739, is the major (wild-type) allele, and the T (X) allele is the minor (rare) allele. To designate the genotype variants of the studied SNV (rs1815739) of the *ACTN3* gene, the following designations were used: homozygous fully functional CC genotype (cytosine/cytosine) or RR (arginine/arginine); heterozygous (intermediate) CT genotype (cytosine/thymine) or RX (arginine/stop codon); homozygous low functional TT genotype (thymine/thymine) or X/X (stop codon/stop codon).

### 2.4. Body Mass Index Assessment

The body mass index (BMI) of athletes and nonathletes was calculated according to the method of Quetelet: m/h^2^, where “m” is body weight in kilograms, and “h” is height in meters.

BMI calculation of less than 18.5 would be underweight. The normal weight BMI numbers range between 18.5 and 24.9. Individuals with BMIs falling between 25 and 29.9 would be classified as overweight. When the BMI calculation ranges between 30 and 39.9, a person is classified as obese. People with a body mass index of more than 40 fall into the category of severely obese.

### 2.5. Body Component Composition Assessment

The assessment of the component composition of the body of the study participants was carried out using multi-frequency bioelectrical impedance analysis (electrical resistance of various parts of the body) InBody-720 (Biospace, Seoul, Republic of Korea). The InBody 720 analyzer uses a four-pole, eight-point tactile electrode system that separately measures arm, torso and leg impedance at three different frequencies (5, 50 and 250 kHz) for each segment. The surface of the hand electrode was placed in contact with each of the five fingers of the hand, while the heels and toes were placed on the foot electrode.

We assessed the composition of the body, including muscle and fat components. Body composition components were calculated as a percentage (%) of the participant’s body weight [20].

### 2.6. Assessment of Physical Performance

The assessment of the sports performance of the study participants (athletes) was carried out using the bicycle ergometry method (test with the maximum load, to failure). The load in the stepped bicycle ergometer test was set by pedaling on a Lode “Corival” (Groningen, The Netherlands) bicycle ergometer with a mechanical braking system. The initial load power was 60 W, and the load was increased by 30 W every 2 min. The maximum power of the performed load (W) was analyzed as an indicator of the functional capabilities of the athlete’s body, integrally reflecting the maximum mobilization capabilities of the body.

### 2.7. Statistical Analysis

All data were analyzed using ISB SPSS version 22.0 software (SPSS Inc., Chicago, IL, USA).

Deviations from Hardy–Weinberg equilibrium (HWE) expectations were tested using a chi-square test. The gene count method checked the distribution of genotypes and allele frequencies. Chi-square test was applied to assess the differences in the distribution of genotypes and allele frequencies between the control group (nonathletes) and comparable subgroups (skaters, sprinters and marathon runners).

The risk of a decrease in physical performance and sports performance in running for short (sprint) and long (marathon) distances was assessed using the odds ratio (odds ratio of 95% confidence interval (CI)). Between-group differences were considered statistically significant at *p*-value < 0.05. Interventional studies involving animals or humans, and other studies that require ethical approval, must list the authority that provided approval and the corresponding ethical approval code.

## 3. Results

### 3.1. Baseline Clinical Characteristics

Relevant baseline clinical characteristics of the participants in this study are presented in Table 2.

The SD subgroup included 40 athletes (mean age 22.1 ± 2.4 years); the LD subgroup included 36 athletes (mean age 22.6 ± 2.7 years); and the control group included 50 healthy volunteers, nonathletes (mean age 21.4 ± 2.7 years). The age of the participants (athletes and nonathletes) varied from 17 to 26 years. The groups were age-matched (*p*-value > 0.05). The participants’ height varied from 166.5 cm to 197 cm for athletes and from 163 cm to 192 cm for nonathletes. The participant weights varied from 59.6 kg to 93 kg for athletes and from 54 to 102 kg for nonathletes. BMI varied from 18.5 to 26.9 kg/m^2^ in athletes and from 17.50 to 29.5 in nonathletes. There were no significant intergroup differences in height (*p*-value > 0.05), weight (*p*-value > 0.05) and BMI (*p*-value > 0.05).

The athletes (SD subgroup and LD subgroup) were additionally analyzed for the indicators characterizing the features of the musculoskeletal system (Table 3). The value of muscle mass varied from 42.69% to 58.44%; the value of fat mass varied from 4.88% to 12.54%. The maximum power of the performed bicycle ergometric load varied from 210 W to 360 W. There were no significant intergroup differences in the studied parameters (*p*-value > 0.05). This should be considered positive and can be explained by the fact that the maximum power completed load is an indicator that integrally reflects maximum mobilization possibilities of the body and the multi-year process of training, if it is rationally organized, is aimed, including increasing the overall performance.

### 3.2. Distribution of Frequencies of Genotypes and Alleles in Athletes with Different Competitive Distances and Individuals in the Control Group

To evaluate the obtained results, we used two models: multiplicative (to estimate the frequency of alleles) and additive (to estimate the frequency of genotypes). In the study of the rs1815739 carriage of the *ACTN3* gene, it was found that the data obtained were consistent with the HWE law: SD subgroup—χ^2^ = 1.25 (*p*-value = 0.264); LD subgroup—χ^2^ = 1.63 (*p*-value = 0.202); and control group—χ^2^ = 2.32 (*p*-value = 0.128). The frequency of the major allele C (R) rs1815739 of the *ACTN3* gene was statistically significantly higher in the SD subgroup compared to the control group: 80% versus 64%, respectively (*p*-value = 0.04) (Table 4).

The frequency of the major allele C (R) rs1815739 of the ACTN3 gene was significantly lower in the LD subgroup compared to the SD subgroup: 80% vs. 59.7%, respectively (*p*-value = 0.05). However, the frequency of the minor allele T(X) rs1815739 was significantly lower in the SD subgroup compared to the LD subgroup: 20.00% vs. 40.30%, respectively (*p*-value = 0.03).

The frequency of the dominant homozygous genotype RR was significantly higher in the SD subgroup compared to the control group: 55.0% vs. 34% (*p*-value = 0.04). The major allele R was significantly associated with competitive distance in the SD subgroup athletes compared to those of the control group (OR = 2.45 (95% CI: 1.02–5.87)) (Table 4). The frequency of the recessive homozygous genotype XX was statistically significantly higher in the LD group compared to the SD subgroup: 11.12% vs. 0% (*p*-value = 0.04). The minor allele X was statistically significantly associated with competitive distance in the LD subgroup compared to the SD subgroup: (OR = 2.70 (95% CI: 1.09–6.68)) (Table 5).

We studied the distribution of allele and genotype frequencies of rs1815739 of the *ACTN3* gene in the athletes depending on their qualification: athletes with different competitive distances (SD and LD) had different qualifications (Table 6 and Table 7).

The frequency of carrying the homozygous RR genotype increases with increasing qualifications in the SD subgroup: 57.14% in the athletes with MSIC and MS qualifications, 40.00% in the group of athletes with CMS qualification and 33.33% in the athletes with category I sports. However, no significant differences were recorded.

There were no significant differences in the frequency of the major allele R and the dominant homozygous genotype RR between athletes of different sports qualifications in the LD subgroup (*p*-value > 0.05).

## 4. Discussion

Our study revealed significant differences in the distribution of the frequencies of genotypes and alleles of this SNV (rs1815739) of the *ACTN3* gene among athletes with different competitive distances (SD and LD) and persons in the control group. Caucasian athletes in cyclic sports in the Southern Urals, using a multiplicative and additive inheritance model, showed a 1.52-time higher OR (95% CI: 1.30–1.77) of high sports performance for sprinters with homozygous genotype 577RR compared with the RX/XX genotypes of the *ACTN3* gene. It can be assumed that at least one copy of the C (R) allele is considered a potentially favorable genetic variation that allows athletes to excel in sprint events. This is evidenced by the fact that the highest frequency of carriers of the major allele R was found in athletes of the SD subgroup, and the homozygous genotype RR prevailed among sprinters with MSIC and MS qualifications. The data obtained are consistent with the studies of other authors, where the highest frequency of carriage of the R allele of the *ACTN3* gene was established in samples of athletes holding world records in sprint races [21], which may be one of the reasons for their superiority in sprint races. For this reason, the *ACTN3* gene has been called the “speed gene” [13], and the RR genotype is considered a potentially favorable genetic variation that allows athletes to excel in sprint disciplines [22].

**Figure 3:**
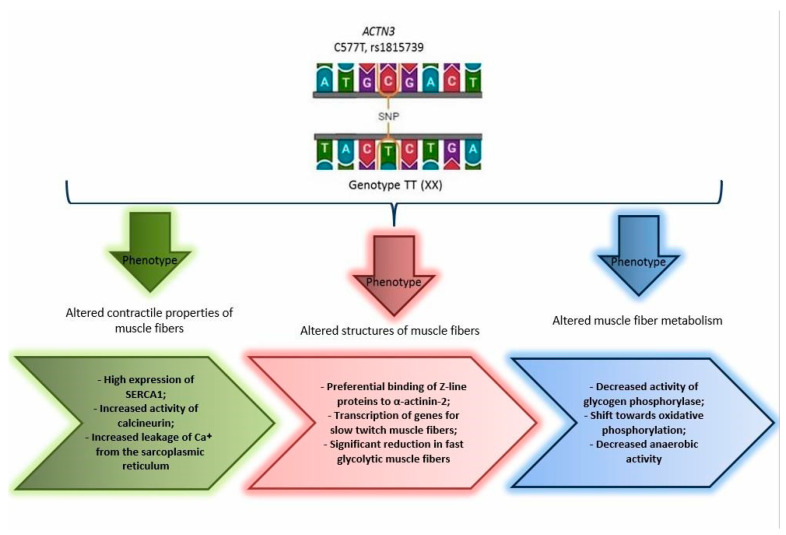
The effect of a genetically determined dysfunction of the actinin-binding protein of fast muscle fibers of type II associated with the single-nucleotide variant rs1815739 of the *ACTN3* gene.

Despite numerous studies that have confirmed an association between the R577 allele of the *ACTN3* gene and sprinter phenotypes in athletes (e.g., sprint time, muscle fiber type), there have been far fewer studies examining the association between the carriage of a minor allele 577X of the *ACTN3* gene and endurance phenotypes [23]. The widespread absence of α-actinin-3 in nearly one-fifth of the world’s population suggests that the role of α-actinin-3 in skeletal muscle may be redundant and not essential for survival [24]. It has been suggested that the homozygous XX genotype increases metabolic efficiency and may even provide a certain advantage in endurance sports, which is associated with altered structural, metabolic and signaling properties of proteins (Figure 3).

Interacting with many muscle proteins, α-actinins perform some signaling and metabolic functions. They interact with calcineurin, which plays an important role in determining the type of muscle fibers [24]. As we know, α-actinin-3 deficiency in the carriers of the homozygous XX genotype increases calcineurin activity (Figure 3). Although calcineurin-dependent pathways are involved in muscle growth and exercise adaptation. The α-actinins also directly interact with soluble signaling factors such as phosphatidylinositol 3-kinase p85, phosphatidylinositol 4,5-bisphosphate and phosphatidylinositol 3,4,5-trisphosphate. All these factors control downstream pathways that regulate several cellular functions, including the PI3K/Akt/mTOR (mechanistic target of rapamycin) signaling cascade that regulates protein synthesis and insulin-like growth-factor-1-mediated muscle fiber hypertrophy. It has also been shown that in the Z-disks of myocytes, α-actinin-2 interacts with atrogin-1, an E3 ubiquitin ligase that is activated during muscle atrophy [20]. Taken together, these data suggest that α-actinin-3 deficiency alters the regulation of muscle mass by modifying key signaling molecules associated with protein synthesis and degradation.

The phenotypic analysis of an *Actn3* knockout mouse model provides some mechanical explanations for the effect of α-actinin-3 deficiency on skeletal muscle performance and performance [24]. The ACTN3 skeletal muscle exhibits a significant decrease in fast twitch type II glycolytic muscle fibers, decreased anaerobic activity and increased oxidative phosphorylation compared to wild-type littermates. This “slowdown” of the metabolic properties of fast glycolytic muscle fibers is due to a decrease in the activity of glycogen phosphorylase [13]. Moreover, α-actinin-3 deficient muscle fibers show an increase in calcineurin activity and changes in contractile characteristics, with an increased rate of decay in calcium signaling caused by increased calcium leakage from the sarcoplasmic reticulum and its reuptake through regulated sarcoplasmic expression, endoplasmic reticulum calcium ATPase 1, calsequestrin and sarcalumenin.

## 5. Conclusions

Indeed, the χ^2^-square test is statistically significant between the SD and LD subgroups and the control group. However, the 95% confidential interval is not statistically significant (0.01–3.81 (XX: SD vs. control) and 0.29–111.06 (XX: SD vs. LD)). On the other hand, the RR genotype is statistically significant (1.00–8.48 (SD vs. Control)). While confirming the value above 1.00, these results support the conclusion that RR is favorable for sprint disciplines, rather than XX is unfavorable for sprinting, in the Caucasian athletes of cyclic sports in the Southern Urals (Chelyabinsk city and Chelyabinsk region, Russia).

## 6. Limitations

Despite numerous studies that have confirmed the relationship between the R577 allele of the rs1815739 *ACTN3* gene and an elite sports status in power sports, there have been far fewer studies on cyclic sports (in particular, track and field athletics and speed skating disciplines). There are also far fewer studies examining the association between the minor allele 577X of the *ACTN3* gene and endurance phenotypes. The strength of our study is in the fact that we studied the association between the carriage of allelic variants rs1815739 of the *ACTN3* gene and both the competitive distance of athletes and their qualifications. Our study has several limitations. The study sample was clearly limited. We studied only athletes of European origin living in the Southern Urals region (Chelyabinsk city and Chelyabinsk region, Russia) and only males. Further analysis of other populations is needed before making strong statements about the predictive role of SNV rs1815739 of the *ACTN3* gene in athletic performance in cyclic sports.

## Figures and Tables

**Table 1:** Criteria for inclusion to and criteria for exclusion from this study.

General Characteristics	Criteria for Inclusion		Criteria for Exclusion
	Group (Athletes)	Group (Nonathletes)	
Residents	Residents of Southern Urals (the city of Chelyabinsk and the Chelyabinsk region, Russia)		Residents of other regions of Russia
Ethnic group	Caucasians		Not Caucasians
Gender	Male		Female
Age	From 17 to 22 years		Under 17
Sports qualification	1st Category, candidate for master of sports (CMS), master of sports (MS), master of sports of international class (MSIC)		No sports qualification
Experience in cyclic sports (speed skating, track and athletics)	5 years or more		No experience in cyclic sports

**Table 2:** Characteristics of the study participants.

General Characteristics	Group 1 (Athletes) (n1 = 76)		Group 2, Control (Nonathletes) (n = 50)	*p*-Value
	SD Subgroup, Short Distances (n1 = 40)	LD Subgroup, Long Distances (n2 = 36)		
Age, years (M ± SD)	22.1 ± 2.4	22.6 ± 2.7	21.4 ± 2.7	>0.05
Height, cm (M ± SD)	183.0 ± 6.43	182.76 ± 5.16	176.39 ± 6.37	>0.05
Weight, kg (M ± SD)	77.01 ± 9.04	70.54 ± 5.11	70.12 ± 9.27	>0.05
BMI according to Quetelet, kg/m^2^ (M ± SD)	22.92 ± 1.94	21.09 ± 1.28	22.52 ± 2.30	>0.05

Note: BMI—body mass index.

**Table 3:** Additional characteristics of cyclic sports athletes.

General Characteristics	SD Subgroup (Athletes, Short Distances) (n1 = 40)	LD Subgroup (Athletes, Long Distances), (n2 = 36)	*p*-Value
The value of the muscle component, % (M ± SD)	50.96 ± 3.71	48.21 ± 2.33	>0.05
The value of the fat component, % (M ± SD)	8.49 ± 1.34	6.98 ± 1.37	>0.05
Maximum power of the executed load, W (M ± SD)	292.11 ± 32.79	297.50 ± 39.52	>0.05

**Table 4:** Allele and genotype of *ACTN3* rs1815739 frequencies.

Alleles, Genotypes	SD Subgroup, Short Distances (*n* = 40)	LD Subgroup, Long Distances (*n* = 36)	Control Group (*n* = 50)
R	32 (80.00%)	43 (59.70%)	62 (62.00%)
X	8 (20.00%)	29 (40.30%)	38 (38.00%)
RR	12 (60.00%)	11 (30.55%)	17 (34.00%)
RX	8 (40.00%)	21 (58.34%)	28 (56.00%)
TT (XX)	0 (0.00%)	4 (11.12%)	5 (10.00%)

**Table 5:** Odds ratio between competitive distance and carriage of minor or major alleles and rs1815739 genotypes of the *ACTN3* gene.

Alleles, Genotypes	χ^2^	*p*-Value	Odds Ratio	95% Confidential Interval
SD Subgroup vs. Control Group				
R	4.20	0.04	2.45	1.02–5.87
X			0.41	0.17–0.98
RR	5.03	0.02	2.91	1.00–8.48
RX			0.52	0.18–1.50
XX			0.20	0.01–3.82
LD Subgroup vs. Control Group				
R	0.33	0.57	0.83	0.45–1.56
X			1.20	0.64–2.24
RR	0.42	0.81	0.78	0.31–1.95
RX			1.10	0.46–2.62
XX			1.14	0.33–6.17
SD Subgroup vs. LD Subgroup				
R	4.78	0.03	0.37	0.15–0.92
X			2.70	1.09–6.68
RR	5.77	0.02	0.29	0.09–0.92
RX			2.10	0.69–6.39
XX			5.68	0.29–111.06

Note: SD—short distances; LD—long distances.

**Table 6:** Allele frequencies and distribution of rs1815739 genotypes of the *ACTN3* gene in athletes—sprinters (sprinting)—with different qualifications.

Alleles, Genotypes	MSIC, MS (*n* = 14)	CMS (*n* = 20)	1st Adult Category (*n* = 6)
R	22 (78.57%)	26 (65.00%)	8 (66.67%)
X	6 (21,43%)	14 (35.00%)	4 (33.33%)
RR	8 (57.14%)	8 (40.00%)	2 (33.33%)
RX	6 (42.86%)	12 (60.00%)	4 (66.67%)
XX	0 (0.00%)	0 (0.00%)	0 (0.00%)

Note: MSIC—master of sports of international class; MS—master of sports; CMS—candidate for master of sports.

**Table 7:** Allele frequencies and distribution of rs1815739 genotypes of the *ACTN3* gene in athletes—long-distance racers—with different qualifications.

Alleles, Genotypes	MSIC, MS (*n* = 15)	CMS (*n* = 13)	1st Adult Category (*n* = 8)
R	17 (56.67%)	16 (61.54%)	10 (62.50%)
X	13 (43.33%)	10 (38.46%)	6 (37.5%)
RR	4 (26.67%)	4 (30.77%)	3 (37.5%)
RX	9 (60.00%)	8 (61.54%)	4 (50.00%)
XX	2 (13.33%)	1 (7.69%)	1 (12.5%)

Note: MSIC—master of sports of international class; MS—master of sports; CMS—candidate for master of sports.

## Data Availability

Not applicable. Data is not available due to confidentiality and ethical restrictions.

## References

[1] Oreshkina I.N., Balberova O.V., Bykov E.V., Sidorkina E.G., Lekontsev E.V. (2019). Construction of training process of skaters in preparatory period based on physical performance. Sci. Notes P.F. Lesgaft Univ..

[2] Balberova O.V. (2021). Candidate genes and single-nucleotide gene variants associated with muscle and tendon injuries in cyclic sports athletes. Pers. Psychiatry Neurol..

[3] Brendan E., Zierath J.R. (2013). Exercise metabolism and the molecular regulation of skeletal muscle adaptation. Cell Metab..

[4] Balberova O.V., Bykov E.V., Medvedev G.V. (2021). Candidate genes associated with athlete skeletal muscle functions regulation. Pers. Psychiatry Neurol..

[5] Jacob Y., Spiteri T., Hart N.H., Anderton R.S. (2018). The potential role of genetic markers in talent identification and athlete assessment in elite sport. Sports.

[6] Ahmetov I.I., Egorova E.S., Gabdrakhmanova L.J., Fedotovskaya O.N. (2016). Genes and athletic performance: Aa update. Med. Sport Sci..

[7] Ahmetov I.I., Donnikov A.E., Trofimov D.Y. (2014). Actn3 genotype is associated with testosterone levels of athletes. Biol. Sport.

[8] Ahmetov I.I., Druzhevskaya A.M., Lyubaeva E.V., Popov D.V., Vinogradova O.L., Williams A.G. (2011). The dependence of preferred competitive racing distance on muscle fibre type composition and ACTN3 genotype in speed skaters. Exp. Physiol..

[9] Maciejewska-Skrendo A., Cięszczyk P., Chycki J., Sawczuk M., Smółka W. (2019). Genetic markers associated with power athlete status. J. Hum. Kinet..

[10] Garton F.C., North K.N. (2016). The Eefect of heterozygosity for the ACTN3 null allele on human muscle performance. Med. Sci. Sports Exerc..

[11] Baltazar-Martins G., Gutiérrez-Hellín J., Aguilar-Navarro M., Ruiz-Moreno C., Moreno-Pérez V., López-Samanes Á., Domínguez R., Del Coso J. (2020). Effect of ACTN3 genotype on sports performance, exercise-induced muscle damage, and injury epidemiology. Sports.

[12] Lee F.X., Houweling P.J., North K.N., Quinlan K.G. (2016). How does α-actinin-3 deficiency alter muscle function? Mechanistic insights into ACTN3, the ‘gene for speed’. Biochim. Biophys. Acta.

[13] Houweling P.J., Papadimitriou I.D., Seto J.T., Pérez L.M., Coso J.D., North K.N., Lucia A., Eynon N. (2018). Is evolutionary loss our gain? The role of ACTN3 p.Arg577Ter (R577X) genotype in athletic performance, ageing, and disease. Hum. Mutat..

[14] Pilegaard H., Saltin B., Neufer P.D. (2003). Exercise induces transient transcriptional activation of the PGC-1alpha gene in human skeletal muscle. J Physiol..

[15] Geography of Genetic Variants Browser. http://www.popgen.uchicago.edu/ggv.

[16] Del Coso J., Hiam D., Houweling P., Pérez L.M., Eynon N., Lucía A. (2019). More than a ‘speed gene’: ACTN3 R577X genotype, trainability, muscle damage, and the risk for injuries. Eur. J. Appl. Physiol..

[17] Alfred T., Ben-Shlomo Y., Cooper R., Hardy R., Cooper C., Deary I.J., Gunnell D., Harris S.E., Kumari M., Martin R.M. (2011). ACTN3 genotype, athletic status, and life course physical capability: Meta-analysis of the published literature and findings from nine studies. Hum. Mutat..

[18] WMA https://www.wma.net/.

[19] Griffiths L.J., Anyim M., Doffman S.R. (2006). Comparison of DNA extraction methods for Aspergillus fumigatususing real-time PCR. J. Med. Microbiol..

[20] Kurose S., Nishikawa S., Nagaoka T., Kusaka M., Kawamura J., Nishioka Y., Sato S., Tsutsumi H., Kimura Y. (2020). Prevalence and risk factors of sarcopenia in community-dwelling older adults visiting regional medical institutions from the Kadoma Sarcopenia Study. Sci. Rep..

[21] Scott R.A., Irving R., Irwin L., Morrison E., Charlton V., Austin K., Tladi D., Deason M., Headley S.A., Kolkhorst F.W. (2010). ACTN3 and ACE genotypes in elite Jamaican and US sprinters. Med. Sci. Sports Exerc..

[22] Papadimitriou I.D., Lucia A., Pitsiladis Y.P., Pushkarev V.P., Dyatlov D.A., Orekhov E.F., Artioli G.G., Guilherme J.P., Lancha A.H., Ginevičienė V. (2016). ACTN3 R577X and ACE I/D gene variants influence performance in elite sprinters: A multi-cohort study. BMC Genom..

[23] Silva M.S., Bolani W., Alves C.R., Biagi D.G., Lemos J.R., da Silva J.L., de Oliveira P.A., Alves G.B., de Oliveira E.M., Negrão C.E. (2015). Elimination of influences of the ACTN3 R577X variant on oxygen uptake by endurance training in healthy individuals. Int. J. Sports Physiol. Perform..

[24] Amorim C.E., Acuña-Alonzo V., Salzano F.M., Bortolini M.C., Hünemeier T. (2015). Differing evolutionary histories of the ACTN3*R577X polymorphism among the major human geographic groups. PLoS ONE.

